# Effective Control of *Schistosoma haematobium* Infection in a Ghanaian Community following Installation of a Water Recreation Area

**DOI:** 10.1371/journal.pntd.0001709

**Published:** 2012-07-17

**Authors:** Karen C. Kosinski, Michael N. Adjei, Kwabena M. Bosompem, Jonathan J. Crocker, John L. Durant, Dickson Osabutey, Jeanine D. Plummer, Miguel J. Stadecker, Anjuli D. Wagner, Mark Woodin, David M. Gute

**Affiliations:** 1 Community Health Program, Tufts University, Medford, Massachusetts, United States of America; 2 Teshie-Accra, Greater Accra Region, Ghana; 3 Parasitology Department, Noguchi Memorial Institute for Medical Research (NMIMR), College of Health Sciences, University Ghana, Legon, Greater Accra Region, Ghana; 4 Department of Environmental Sciences and Engineering, Gillings School of Global Public Health, University of North Carolina at Chapel Hill, Chapel Hill, North Carolina, United States of America; 5 Department of Civil and Environmental Engineering, Tufts University, Medford, Massachusetts, United States of America; 6 Department of Civil and Environmental Engineering, Worcester Polytechnic Institute, Worcester, Massachusetts, United States of America; 7 Department of Pathology, The Sackler School of Biomedical Sciences, Tufts University School of Medicine, Boston, Massachusetts, United States of America; 8 Department of Epidemiology, University of Washington, Seattle, Washington, United States of America; Jiangsu Institute of Parasitic Diseases, China

## Abstract

**Background:**

Urogenital schistosomiasis caused by *Schistosoma haematobium* was endemic in Adasawase, Ghana in 2007. Transmission was reported to be primarily through recreational water contact.

**Methods:**

We designed a water recreation area (WRA) to prevent transmission to school-aged children. The WRA features a concrete pool supplied by a borehole well and a gravity-driven rainwater collection system; it is 30 m^2^ and is split into shallow and deep sections to accommodate a variety of age groups. The WRA opened in 2009 and children were encouraged to use it for recreation as opposed to the local river. We screened children annually for *S. haematobium* eggs in their urine in 2008, 2009, and 2010 and established differences in infection rates before (2008–09) and after (2009–10) installation of the WRA. After each annual screening, children were treated with praziquantel and rescreened to confirm parasite clearance.

**Principal Findings:**

Initial baseline testing in 2008 established that 105 of 247 (42.5%) children were egg-positive. In 2009, with drug treatment alone, the pre-WRA annual cumulative incidence of infection was 29 of 216 (13.4%). In 2010, this incidence rate fell significantly (p<0.001, chi-squared) to 9 of 245 (3.7%) children after installation of the WRA. Logistic regression analysis was used to determine correlates of infection among the variables age, sex, distance between home and river, minutes observed at the river, low height-for-age, low weight-for-age, low Body Mass Index (BMI)-for-age, and previous infection status.

**Conclusion/Significance:**

The installation and use of a WRA is a feasible and highly effective means to reduce the incidence of schistosomiasis in school-aged children in a rural Ghanaian community. In conjunction with drug treatment and education, such an intervention can represent a significant step towards the control of schistosomiasis. The WRA should be tested in other water-rich endemic areas to determine whether infection prevalence can be substantially reduced.

## Author Summary

Urogenital schistosomiasis is a disease caused by the parasite *Schistosoma haematobium*; it is often characterized by bloody urine and tends to disproportionately affect school-aged children in rural tropical regions. The parasite is transmitted via skin contact with surface water that is contaminated by human waste. The disease was endemic in Adasawase, a rural Ghanaian community, in 2007. Transmission occurred mainly through recreational water contact. We collaborated with community members to design a water recreation area (WRA) featuring a concrete pool supplied by a borehole well and a rainwater collection system. We opened the pool in 2009 and local officials encouraged children to use the WRA for recreation. We screened local children annually (2008, 2009, 2010) for *S. haematobium* infection. After each screening, children were treated with praziquantel and rescreened. Baseline testing in 2008 established that at least 105 of 247 (42.5%) children were infected. In 2009, 29 of 216 (13.4%) children were infected, reflecting annual cumulative incidence. In 2010, a significantly smaller percentage of children (9 of 245, 3.7%) were infected. We conclude that the WRA effectively reduced infection in Adasawase, and that it should be tested in other water-rich endemic areas.

## Introduction

### Urogenital Schistosomiasis

Schistosomiasis is a neglected tropical disease caused by parasitic trematodes of the genus *Schistosoma*. In 2006, Steinmann et al. estimated that globally, 207 million people live with schistosomiasis [1], but later estimates by King suggest that the number is between 391 and 587 million people [2]. Morbidity may result from chronic or acute infection and may be independent of worm burden [3]. Hematuria is not exclusive to *S. haematobium* infection but it is estimated that in West Africa, over 15% of the population experiences hematuria at any given time [4]. Studies reviewed by Mbabazi et al. strongly point to *S. haematobium* infection as a risk factor for contracting HIV, particularly for women [5]. As described below, there are a variety of risk factors for contracting schistosomes, and correspondingly, a variety of options to control morbidity and transmission.

### Risk of Schistosoma haematobium Infection

Risk factors for *S. haematobium* infection tend to be location-specific; they may include age, sex, occupation, water contact practices, socioeconomic status, and distance to safe and unsafe water sources. Age and sex are two commonly studied infection risk factors. The prevalence of hematuria and *S. haematobium* eggs in urine tends to increase throughout childhood and peak between the ages of 10 and 20 as a function of increasing contact with infested water [6]–[10]. Decreases in worm burden after adolescence may be due to changes in immunity and/or behavior [10]. Males often have higher prevalences of infection and higher mean egg counts than do females [7], [8], but this is not always the case [11]–[13]. Sex-based differences in infection are thought to result from behavior differences.

Previous infection with schistosomes is a complex risk factor and may predict likelihood of current infection. Previous infection may indicate behaviors that increase the subsequent risk of reinfection, but could also be associated with a relatively high likelihood of recent treatment with praziquantel, the drug of choice to kill schistosomes [7]. Finally, previous infection may correlate with acquired immunity [10].

Clothes washing, water collection, swimming/bathing, and fishing have all been identified as risk factors for schistosome infection [7], [14] with varying results. For example, Hammad et al., Handzel et al., and Stothard et al. found correlations between water contact and infection [7], [14], [15]; other studies showed that proximity to contaminated surface water is a relevant factor [12], [14]. In contrast, Satayathum et al. working in Kenya and Pereira et al. working in Brazil did not find correlations between water contact and infection [11], [16].

### Control of Urogenital Schistosomiasis

In 1993, the World Health Organization (WHO) stated that control of schistosomiasis should be accomplished within the context of the existing primary health care system, and that a long-term commitment (10 to 20 years) to this goal is necessary [17]. Control programs can be broadly categorized into transmission control and/or morbidity control initiatives. Control options were recently reviewed by King [2]. Current control methods are the following: mass drug administration (MDA); water, sanitation and hygiene programs; education and behavior change programs; and occasionally, snail control. There is no single solution that is appropriate for every setting. Stothard et al. argue for the need to address *S. haematobium* transmission via improved access to clean water, education, and behavior change [15]. Satayathum et al. [11] determined that annual treatment of egg-positive school-aged children in Kenya could not reduce infection prevalence below 14% between 1984 and 1992. After seven years of intensive health education in Senegal, knowledge of *S. mansoni* infection, transmission, symptoms, and treatment remained very low among both children and adults [18]. The authors concluded that community-driven control would be more effective than a vertical approach and behavior change may not occur when individuals lack access to infrastructure. In China, there is evidence that integrated control is highly effective in controlling *S. japonicum* [19], [20].

Our goal was to assess *S. haematobium* infection rates in the absence and presence of a water recreation area (WRA) designed to reduce water contact and *S. haematobium* annual cumulative incidence. We focused on *S. haematobium* infections in Adasawase, Ghana where infection is typically transmitted via recreational contact with the Tini River. The location was selected based on a relatively high prevalence of *S. haematobium* infection as reported by the Chief of Adasawase in December 2007. The main objective of our study was to test the hypothesis (chi-squared analysis) that the annual cumulative incidence of *S. haematobium* infection among a population of schoolchildren would decrease in the presence of a WRA. The study team was invited by the Chief of Adasawase to test this hypothesis by assessing annual cumulative incidence of *S. haematobium* infection before and after WRA installation.

## Methods

### Ethics Statement

The study protocol was approved by the Social, Behavioral, and Educational Research Institutional Review Board (IRB) of Tufts University and the IRB of the Noguchi Memorial Institute for Medical Research (NMIMR) in Accra, Ghana. The Chief of Adasawase and the head of each school provided written permission to conduct the study protocol. The Chief and the school heads communicated with parents and community members about the nature of the study. As part of this process, a number of public meetings were held by the Chief of Adasawase and the Council of Elders. Senior study team members were present at these meetings and answered questions about the study protocol as posed by parents, guardians, and potential participants. The IRBs of both Tufts University and NMIMR approved the study protocol, which employed verbal informed consent from parents/guardians, verbal informed consent from adult participants (≥18 years), and verbal assent from school-aged participants. A waiver of documentation of informed consent was approved by both IRBs. Only individuals <18 years whose parent/guardian provided verbal consent were enrolled in the study. This protocol was considered appropriate in a town where parents/guardians have historically expressed concern about signing formal paperwork for a non-invasive procedure (e.g., providing urine samples) and where schistosomiasis prevalence is high but treatment options are few. Each study participant provided verbal assent (or verbal consent for participants ≥18 years) in the presence of several witnesses; this verbal assent was obtained prior to the collection of each urine sample and at each praziquantel treatment encounter administered by Ghana Health Services staff.

### Study Design

**Figure 1:**
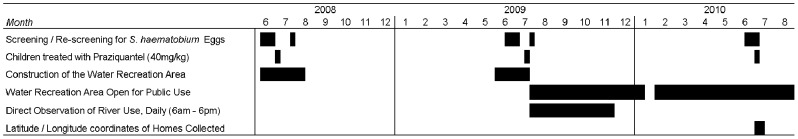
Main study activities carried out in Adasawase, Ghana (2008–2010).

The study described here was conducted over three years (Figure 1). In 2008, infection prevalence for *S. haematobium* in Adasawase was quantified, all children were treated with praziquantel by Ghana Health Services as per WHO Guidelines [21], and construction of the WRA began. The design, construction, and operation methods were all chosen for sustainability and ability to be replicated in other settings. The WRA is described in detail elsewhere [22]. Briefly, the WRA is a concrete pool fed by rainwater and hand-pumped groundwater; it is approximately 30 square meters and is divided into shallow and deep sections to meet the needs of children in a variety of age groups. In 2009, (re)infection in the community was quantified in the absence of the WRA and children were again treated with praziquantel. Directly after the WRA was opened for public use in July 2009, water contact at the local river was observed. In 2010, reinfection was quantified after the WRA had been used for one year; egg-positive children were specifically targeted for treatment with praziquantel, but any child who wished to take praziquantel was treated [21].

For data analysis, study participants were separated into five cohorts based on age, school enrollment, screening status, treatment status, and whether previous infection status was known (P.I.S.K.) (Table 1). The 2008 cohort was chosen based on age, school enrollment, and participation in three screenings. The 2009 and 2010 cohorts were both chosen based on the following: age; school enrollment; participation in three or more screenings in the relevant year; treatment with praziquantel in the previous year; and negative *S. haematobium* status in the previous year at baseline. The 2009-P.I.S.K. and 2010-P.I.S.K. cohorts are made up of children with these same characteristics, in addition to the criteria that they were screened three or more times in the previous year and their previous infection status was known (P.I.S.K.).

**Table 1:** Criteria by which study participants in were included in each cohort.

	Criteria for Inclusion in Data Analysis				
	2008	2009	2009-P.I.S.K.*	2010	2010-P.I.S.K.
8 years of age or older	X	X	X	X	X
Enrolled in school in June of screening year	X	X	X	X	X
Screened 3+ times in June/July of screening year	X	X	X	X	X
Screened 3+ times in June/July of previous year			X		X
Treated with praziquantel in previous year		X	X	X	X
Screened 1 time after previous praziquantel treatment and found negative **and/or** tested negative on 3+ screenings prior to praziquantel in previous year		X	X	X	X
Total number of participants	247	216	133	245	186

*: P.I.S.K. = Previous Infection Status Known.

### Study Population

Adasawase has a population of approximately 2,000 residents. S. haematobium infection was monitored in residents 8 to 22 years of age who were enrolled in one of three schools (one junior high school, two primary schools) in Adasawase as of June 2008, June 2009, and/or June 2010. The number, percentage and age of children screened at least three times in any given year are shown in Table 2. Not all of these children were previously treated with praziquantel and re-screened; thus, the number of study participants whose data were used in 2009 and 2010 is smaller than the number shown in Table 2.

**Table 2:** Children screened at least three times.

	2008 - GIRLS			2009 - GIRLS			2010 - GIRLS		
Birth Year	Screened 3+ Times	Total Number Enrolled	(%) Screened 3+ Times	Screened 3+ Times	Total Number Enrolled	(%) Screened 3+ Times	Screened 3+ Times	Total Number Enrolled	(%) Screened 3+ Times
2002							21	21	100.0
2001				14	21	66.7	20	23	87.0
2000	0	10	0.0	18	24	75.0	14	19	73.7
1999	16	37	43.2	29	34	85.3	24	26	92.3
1998	10	22	45.5	15	21	71.4	17	20	85.0
1997	24	35	68.6	27	30	90.0	22	25	88.0
1996	15	24	62.5	13	18	72.2	13	15	86.7
1995	14	19	73.7	10	15	66.7	7	7	100.0
1994	9	13	69.2	8	13	61.5	8	9	88.9
1993	7	8	87.5	7	8	87.5	3	6	50.0
1992	17	24	70.8	6	8	75.0	4	5	80.0
1991	5	9	55.6	1	2	50.0			
1990	2	2	100.0						
**Total**	119	203	58.6	148	194	76.3	153	176	86.9

	2008 - BOYS			2009 - BOYS			2010 - BOYS		
Birth Year	Screened 3+ Times	Total Number Enrolled	(%) Screened 3+ Times	Screened 3+ Times	Total Number Enrolled	(%) Screened 3+ Times	Screened 3+ Times	Total Number Enrolled	(%) Screened 3+ Times
2002							15	18	83.3
2001				10	19	52.6	27	33	81.8
2000	0	9	0.0	15	19	78.9	20	22	90.9
1999	18	42	42.9	32	39	82.1	32	34	94.1
1998	16	34	47.1	22	32	68.8	23	27	85.2
1997	9	18	50.0	15	19	78.9	17	19	89.5
1996	18	26	69.2	13	22	59.1	15	19	78.9
1995	31	48	64.6	32	44	72.7	34	39	87.2
1994	16	18	88.9	10	16	62.5	9	14	64.3
1993	4	18	22.2	7	14	50.0	2	2	100.0
1992	10	31	32.3	7	13	53.8	10	12	83.3
1991	8	19	42.1	2	3	66.7	3	3	100.0
1990	3	3	100.0	1	1	100.0			
1989	1	2	50.0						
1988	0	1	0.0	0	1	0.0	0	1	0.0
**Total**	134	269	49.8	166	242	68.6	207	242	85.5

### Parasitological Data

*S. haematobium* infection prevalence was quantified in June of 2008 and annual cumulative incidence in June/July of 2009 and 2010. Infection status was determined by urine filtration and subsequent microscopy for identification of *S. haematobium* eggs. Urine was collected in conical 50 mL tubes between 10:00 and 14:00 hours from children who were present at school. Schools were visited up to seven (2008) or nine (2009, 2010) times to request samples from each study participant. Once a child provided three (2008) or four (2009, 2010) samples on different days, (s)he was not asked for additional samples. We screened each child multiple times to improve the accuracy of prevalence and annual cumulative incidence estimates [23]. Data from children who provided at least three samples are presented here. A flow chart of the study design is available upon request.

Urine samples were tested for *S. haematobium* eggs via filtration through Nucleopore membranes (25 mm diameter, 12.0 µm pore size; Sterlitech Corporation, Kent, Washington). Urine was shaken, drawn into a 10 mL syringe, and then discharged through a new Nucleopore membrane. Filtered urine was discarded. In this study, samples were tested either by extracting a 10 mL sub-sample of urine and filtering it, or by filtering the entire urine volume, which is a slightly more sensitive method. The first urine sample each year that a child submitted was tested by filtering a 10 mL sub-sample of urine. Membranes were removed from filter holders with forceps and placed egg-side-down on glass slides and examined under 100× magnification. For every sample, all *S. haematobium* eggs on each membrane were counted by the same experienced laboratory technician from the Noguchi Memorial Institute for Medical Research. To standardize results between 10 mL and full volume filtration methods, only urine samples with at least 1 egg/10 mL urine were considered positive in this study. Data were reduced to a binary score of positive or negative for *S. haematobium* eggs for analysis.

### Directly Observed Behavioral Data

A single community member from Adasawase was trained to directly observe behavior at the Tini River, the only recreational water contact site used by the community. The observer is a resident of the town and had established rapport with children, teachers, and parents in Adasawase. He answered questions about the study if asked and did not record information about anyone who did not wish to be observed (no such requests were made). He observed the river from 6:00 to 18:00 hours for 14 days between July 5 and July 31, 2009. He also observed the river from 6:00 to 18:00 hours 7 days per week (84 hours/week) from 1 August to 30 November 2009. Use of the river after 18:00 hours was very rare (data not collected). The following data were collected for each school-aged person who visited the river: name, age, school attended, school class, time of day, minutes in contact with river water, and activities performed (swimming, washing/bathing, water collection, and washing of clothing or utensils).

### Anthropometric Data

Each child's height (centimeters) and weight (kilograms) were measured. For height measurements, children stood barefoot against a stadiometer. In 2008 and 2009, height was recorded to the nearest 0.10 cm; in 2010, height was recorded to the nearest 0.50 cm. A digital scale (2008, 2009) or mechanical scale (2010) was used to record the child's weight to the nearest 0.1 kg. Children wore school uniforms when they were measured. Body mass index was calculated as BMI = weight (kg)/height (m^2^).

A child's status as having low height-for-age and/or low BMI-for-age was determined based on the “Simplified Field Tables” from the WHO [24], [25]. A child's status as having low weight-for-age was determined based on the Growth Charts from the United States Centers for Disease Control and Prevention [26]. The birth days and months of children were unknown because the children were not able to self-report this information. Birth years were obtained from school records. The cutoff point (inclusive) for low height-for-age was -2 standard deviations (SD) for the child's age in years plus 6 months. The cutoff point (inclusive) for low weight-for-age was the fifth percentile for the child's age in years plus 6 months. The cutoff point for low BMI-for-age was the third percentile for the child's age in years plus 6 months, inclusive.

### Residential and Spatial Data

In July 2010, a spatial plan of Adasawase was created to determine the locations of homes of children enrolled in the study. One team member from Adasawase located houses, explained the study to participants, obtained verbal consent to participate in the study, and asked questions about school-aged individuals in each home. The other team member used a handheld global positioning system (GPS) unit (Garmin GPS 60 Portable Navigator, Garmin, Ltd.) to record the latitude and longitude coordinates of homes and well-known town landmarks; he also recorded data in a field notebook. Household members were verbally asked to provide the name, age, sex, grade level, and school of each school-aged child in the household. These data were recorded and manually matched to the child's parasitological and demographic data. When an exact match of information was not found, the relevant household was revisited and follow-up questions were asked to rectify any discrepancies.

GIS layers were constructed by digitizing satellite imagery against the latitude and longitude coordinates of landmarks collected with the handheld GPS unit. Once the satellite image was georectified (World Geodetic System 1984, 30N), walking paths, roads, surface water, and points of interests were manually digitized from the image. House locations were imported into ArcGIS (version 9.3.1) from the handheld GPS unit. The objective was to determine the locations of houses with respect to the Tini River.

In the analysis of the spatial data, several simplifying assumptions were made. As a measure of distance, we employed the linear distance between homes and the main swimming point in Tini River, as opposed to the walking distance. The distance between a child's home and the Tini River was calculated in meters; the data were then reduced to an ordinal number corresponding to 1 = close (<500 m), 2 = medium distance (≥500 m and ≤1,000 m), and 3 = far (>1,000 m). These cutoff points were assigned by the research team based on the size of the community. In addition, we assumed that children reported to live at a particular home in 2010 resided in that same home in 2008 and 2009.

### Logistic Regression

Logistic regression (LR) can be used for prediction [27], [28], hypothesis testing, or the determination of the statistical significance of covariates [7], [27]. Here, LR was used to assess the significance of covariates. Parasitological (hematuria and egg data), behavioral, anthropometric, and spatial data were double-entered into SPSS 14.0 (SPSS Inc., Chicago, IL). Three separate univariable analyses of potential risk factors for infection with *S. haematobium* were conducted to identify risk factors that were likely to be significant in multivariable models (results not shown). The dichotomous outcome variable in each case is *S. haematobium* infection status in 2008, 2009, and 2010. After univariable analysis, potential predictor variables with respect to multivariable LR were identified based on whether or not they were significant (p<0.05) or marginally significant (0.05≤p<0.10).

Potential covariates were tested via LR analysis to determine whether or not their association with infection status was statistically significant. For 2008 infection status, variables tested included the following: age (in years), sex, the distance between a child's home and the Tini River (in meters), and the number of minutes a child was observed to be in contact with the river in 2009. These same risk factors were considered for 2009, in addition to the variable ‘previous infection status’ (either positive or negative). Note that observation of behavior in 2009 took place after children were screened in 2008 and 2009; thus, behavior in 2009 is used as a proxy for behavior that occurred prior to the 2008 and 2009 screenings. A final model for each LR analysis was chosen once all variables in the model were either statistically significant or biologically plausible *and* marginally significant.

## Results and Discussion

Annual cumulative infection incidence decreased significantly in girls and boys in the presence of a WRA. Risk factors for infection also changed significantly during the course of the study. The baseline prevalence of *S. haematobium* infection in 2008 and the annual cumulative incidence of infection in 2009 and 2010 are shown in Table 3. In 2008, data from all children in the cohort 2008 (see Table 1) were used to calculate cross-sectional prevalence. The criterion of participating in at least three screenings was applied in each year of the study and thus infection results in 2008, 2009, and 2010 were ascertained uniformly.

**Table 3:** Prevalence (2008) and annual cumulative incidence of *S. haematobium* infection (2009 and 2010).

	2008 - GIRLS			2009 - GIRLS			2010 - GIRLS		
Birth Year	*S. haematobium* Egg Positive	Screened 3+ Times	(%)	*S. haematobium* Egg Positive	Screened 3+ Times	(%)	*S. haematobium* Egg Positive	Screened 3+ Times	(%)
2002							0	6	0.0
2001				0	1	0.0	0	16	0.0
2000				1	7	14.3	1	9	11.1
1999	1	15	6.7	2	22	9.1	0	20	0.0
1998	1	9	11.1	1	9	11.1	0	11	0.0
1997	9	24	37.5	4	19	21.1	1	15	6.7
1996	6	15	40.0	0	11	0.0	0	7	0.0
1995	5	14	35.7	1	10	10.0	0	7	0.0
1994	3	9	33.3	1	6	16.7	0	8	0.0
1993	3	7	42.9	1	5	20.0	0	3	0.0
1992	9	17	52.9	0	5	0.0	0	3	0.0
1991	2	5	40.0	0	1	0.0			
1990	1	2	50.0						
**Total**	40	117	34.2	11	96	11.5	2	105	1.9

	2008 - BOYS			2009 - BOYS			2010 - BOYS		
Birth Year	*S. haematobium* Egg Positive	Screened 3+ Times	(%)	*S. haematobium* Egg Positive	Screened 3+ Times	(%)	*S. haematobium* Egg Positive	Screened 3+ Times	(%)
2002							0	2	0.0
2001				0	1	0.0	0	16	0.0
2000				1	6	16.7	0	12	0.0
1999	10	18	55.6	5	28	17.9	2	26	7.7
1998	6	16	37.5	3	19	15.8	2	19	10.5
1997	4	9	44.4	3	11	27.3	0	14	0.0
1996	9	15	60.0	1	9	11.1	1	13	7.7
1995	15	30	50.0	4	24	16.7	2	22	9.1
1994	9	16	56.3	1	9	11.1	0	7	0.0
1993	2	4	50.0	0	5	0.0	0	2	0.0
1992	4	10	40.0	0	7	0.0	0	5	0.0
1991	4	8	50.0				0	2	0.0
1990	2	3	66.7	0	1	0.0			
1989	0	1	0.0						
**Total**	65	130	50.0	18	120	15.0	7	140	5.0

In 2008, 34.2% (n = 40/117) of girls and 50.0% (n = 65/130) of boys (cohort = 2008) were positive for *S. haematobium* eggs. In 2009, 11.5% (n = 11/96) of girls and 15.0% (n = 18/120) of boys were infected with *S. haematobium* (cohort = 2009), reflecting annual cumulative incidence in the absence of the WRA. In 2010, 1.9% (n = 2/105) of girls and 5.0% (n = 7/140) of boys were positive for *S. haematobium* eggs (cohort = 2010), reflecting annual cumulative incidence in the presence of the WRA. Annual cumulative incidence of *S. haematobium* infection was higher in the absence of the WRA than in the presence of the WRA [(Girls: χ*^2^* = 7.57, df = 1, p<0.01), Boys: χ*^2^* = 7.43, df = 1, p<0.01)].

### Spatial Plan of Adasawase

Spatial data can be collected and used to characterize patterns of *S. haematobium* infection, water contact behavior, and environmental factors within communities. Because risk factors for infection vary among and within communities, additional studies are needed to document the spatial heterogeneity of schistosome infection and to better characterize significant risk factors other than climate and terrain [29], [30]. For example, Clennon et al. mapped home and water contact locations in Kenya via very high resolution (1 to 4 m^2^) remotely-sensed imagery and concluded that children under 6 who live near water contact sites may have more exposure to contaminated water and thus may develop immunity to reinfection earlier than children who live farther away [31].

We created a spatial plan of Adasawase using GIS data collected in 2010 (see reference 22 for figure). The home locations for 71 out of 254 children in the 2008 cohort were available in 2010. The home locations for 86 out of 220 children were available for the 2009 cohort, and for 117 out of 246 children in the 2010 cohort. The home locations for the rest of the children in each cohort remain unknown; these children were not reported to be living at any of the households visited by the study team, which may indicate that they live outside the town, they have moved away from the town, the individual reporting house resident names did not consider them a ‘resident’, or the name given in the household is not the name the child uses in school.

### Risk Factors of Infection

Potential risk factors for developing urogenital schistosomiasis were evaluated via LR analysis by Hammad et al., Nsowah-Nuamah et al., and Clennon et al. [7], [32], [33]. Here, a univariable analysis of risk factors was conducted (not shown) and then LR analysis was performed. Prior to the intervention (2009 model only), variables that were associated (p<0.05) with infection include: the distance between a child's home and the Tini River, the number of minutes observed using the river, and previous infection status. Post-intervention in 2010, none of the variables remained significant (p<0.05) as predictors of infection status.

### Directly Observed Behavior at the Tini River

Children who were egg-positive for *S. haematobium* in any given year were more likely than their sex-matched peers to (a) use the river and (b) use the river for longer periods (Table 4). The number of “contacts per person” refers to the number of different occasions on which a person used the river. Directly observed behavior in 2009 was considered a proxy for behavior in 2008, 2009, and 2010.

**Table 4:** Use of the Tini River by girls and boys between 5 July and 30 November 2009.

	Use of the Tini River - GIRLS					
	2008		2009		2010	
	*S. haematobium*		*S. haematobium*		*S. haematobium*	
	Positive	Negative	Positive	Negative	Positive	Negative
Total number of children	40	77	11	85	2	103
Number of children observed using the Tini River in 2009	8/40 (20.0%)	4/77 (5.2%)	5/11 (45.5%)	7/85 (8.2%)	1/2 (50.0%)	13/103 (12.6%)
Total time the Tini River was used in 2009 (min)	2245	560	2140	1075	475	3745
Total contact time per person, Range (min)	20 to 685	20 to 475	20 to 685	20 to 475	475	20 to 685
Mean contact time per person who used the Tini River (min ± s.d.)	281±254	187±251	428±268	154±153	475	288±240
Range of number of contacts per person who used the Tini River (contacts)	2 to 41	1 to 32	2 to 41	1 to 36	32	1 to 41
	Use of the Tini River - BOYS					
Total number of children	65	65	18	102	7	133
Number of children observed using the Tini River in 2009	12/65 (18.5%)	7/65 (10.8%)	9/18 (50.0%)	13/102 (12.7%)	1/7 (14.3%)	32/133 (24.1%)
Total time the Tini River was used in 2009 (min)	3889	870	3561	1820	330	5846
Total contact time per person, Range (min)	5 to 832	10 to 345	110 to 832	5 to 585	330	5 to 832
Mean contact time per person who used the Tini River (min ± s.d.)	324±252	124±128	396±236	140±157	330	183±197
Range of number of contacts per person who used the Tini River (contacts)	1 to 53	1 to 19	9 to 53	1 to 52	23	1 to 53

### Baseline *S. haematobium* Infection

A logistic regression model was developed for the dichotomous outcome variable *S. haematobium* infection status in 2008. Variables considered in the model include: age, sex, distance between home and the Tini River, minutes observed at the river, low height-for-age, low weight-for-age, and low BMI-for-age. Sex and minutes observed at the river were significant in the model (Table 5); age is of marginal significance but is retained in the model for comparison with other studies. The distance between a child's home and the Tini River may have been a relevant factor for infection, but the sample size for this variable was relatively small (n = 71) and it was not significant in the final model. The model correctly identified 81.7% of negative children and 33.3% of positive children, indicating that while relevant, not all variability in infection status is captured by these explanatory factors. The final model was chosen based on the significance of each explanatory variable, on biological plausibility, and on a non-significant Hosmer and Lemeshow goodness-of-fit test (p = 0.385). The model shows that for every one additional year of age, risk of infection increases by a factor of 1.112 (95% CI: 1.002–1.235); males were nearly 1.9 times more likely than females to be infected (95% CI: 1.108–3.198); and for every additional hour spent in contact with the Tini River, risk of infection increases by 1.349 (95% CI: 1.062–1.613).

**Table 5:** Risk factors associated with *S. haematobium* infection in 2008, as assessed by egg count.

							95% CI	
Variable	n	B	S.E.	Wald	Sig.	OR	Lower	Upper
Age	247	0.106	0.053	3.961	0.047	1.112	1.002	1.235
Sex (Female)	247	0.633	0.270	5.472	0.019	1.882	1.108	3.198
Minutes Observed at River, Jul–Nov 2009	247	0.005	0.002	7.593	0.006	1.005	1.001	1.008
Constant		−2.121	0.718	8.732	0.003	0.120		

### Pre-WRA *S. haematobium* Infection

Two different logistic regression models were developed for the dichotomous outcome variable “*S. haematobium* infection status in 2009”. In the first model (Table 6), variables considered include: age, sex, distance between home and the Tini River, minutes observed at the river, low height-for-age, low weight-for-age, low BMI-for-age, and 2008 infection status. To assess the significance of the variable ‘2008 infection status’, only data from children in the group 2009-P.I.S.K. (Table 1) were considered (n = 133). The number of minutes observed at the river (OR = 1.006, 95% CI: 1.002–1.010) and 2008 infection status (OR = 9.422, 95% CI: 1.932–45.938) were the only variables significantly associated with *S. haematobium* infection in 2009. The model correctly identified 99.1% of negative children and 38.9% of positive children (Hosmer and Lemeshow = 0.691). The final model was chosen based on the significance of each explanatory variable and on biological plausibility.

**Table 6:** Risk factors associated with *S. haematobium* infection in 2009.

							95% CI	
Variable	n	B	S.E.	Wald	Sig.	OR	Lower	Upper
Minutes Observed at River, Jul–Nov 2009	133	0.006	0.002	9.988	0.002	1.006	1.002	1.010
2008 Infection Status (Negative)	133	2.243	0.808	7.700	0.006	9.422	1.932	45.938
Constant		−3.834	0.743	26.598	0.000	0.022		

A second logistic regression model was developed for the dichotomous outcome variable “*S. haematobium* infection status in 2009” that did not consider 2008 infection status as an explanatory variable (Table 7). In the second model, variables considered include: age, sex, distance between home and the Tini River, minutes observed at the river, low height-for-age, low weight-for-age, and low BMI-for-age. The number of minutes observed at the river was the only factor significantly associated with infection in 2009 (OR = 1.007, 95% CI: 1.004–1.011). Sex was not significant (p = 0.564), but was retained in the model for comparison. The model correctly identified 98.9% of negative children and 31.0% of positive children (Hosmer and Lemeshow = 0.108). The final model was chosen based on the significance of each explanatory variable and on biological plausibility.

**Table 7:** Risk factors associated with *S. haematobium* infection in 2009; variable ‘2008 Infection Status’ not considered.

							95% CI	
Variable	n	B	S.E.	Wald	Sig.	OR	Lower	Upper
Minutes Observed at River, Jul–Nov 2009	216	0.007	0.002	21.324	0.000	1.007	1.004	1.011
Sex (Female)	216	0.271	0.469	0.333	0.564	1.311	0.523	3.284
Constant		−2.522	0.381	43.914	0.000	0.080		

### Post-WRA *S. haematobium* Infection

Nine children were infected with *S. haematobium* following construction of the WRA. The annual cumulative incidence rate (3.7%) in the presence (2009 to 2010) of the WRA was significantly lower than the annual cumulative incidence rate (13.4%) in the absence (2008 to 2009) of the WRA (χ^2^ = 14.44, df = 1, p<0.001). Because of the small number of infected children in 2010, a well-powered logistic regression analysis to determine risk factors was not feasible. Instead, the potential risk factors associated with infection are presented for each child (Table 8). Of the infected children, 7 of 9 were male; all were between the ages of 11 and 15 years; nearly half (4/9) had a history of previous *S. haematobium* infection; and 8 of 9 children had at least one potential indicator of malnutrition (low height-for-age, low weight-for-age, or low BMI-for-age). These risk factors suggest demographic and behavioral characteristics that may be associated with infection. However, it is not possible to conclusively state why each child was infected.

**Table 8:** Descriptive characteristics of nine *S. haematobium*-positive children in 2010, post-intervention.

	*S. haematobium* positive children in 2010								
Assigned Case Number	1	2	3	4	5	6	7	8	9
Age as of 2010	10	12	11	15	15	12	13	14	11
Sex (F/M)	F	M	M	M	M	M	F	M	M
Distance between home and the Tini River	Med.	Far	Unknown	Med.	Unknown	Far	Close	Far	Unknown
Total minutes observed using the river	0	0	0	330	0	0	475	0	0
% of days attended school in 2010	0.87	0.97	0.59	0.87	0.91	0.93	0.92	0.96	0.97
New resident of Adasawase as of 2009	1	0	0	1	1	0	0	0	0
Number of times screened in 2010	4	3	3	4	4	4	4	4	4
Low height-for-age in 2009	n/a	1	1	1	1	n/a	n/a	1	0
Low height-for-age in 2010	1	1	1	1	1	1	0	1	0
Low weight-for-age in 2009	1	0	1	1	1	1	1	1	0
Low weight-for-age in 2010	1	1	1	1	0	1	0	1	0
Low BMI-for-age in 2009	n/a	0	0	1	0	n/a	n/a	0	0
Low BMI-for-age in 2010	0	0	0	0	0	1	0	0	0
Egg-positive in 2008	n/a	No	Yes	n/a	n/a	Yes	No	No	Yes
Number of times screened in 2009	4	4	4	3	4	4	4	4	4
Egg-positive in 2009	Yes	No	No	No	No	Yes	Yes	No	No
Treated in 2009	Yes	Yes	Yes	Yes	Yes	Yes	Yes	Yes	Yes
Tested after praziquantel in 2009	Yes	No	No	No	No	Yes	Yes	No	No
Egg-positive after praziquantel in 2009	No	n/a	n/a	n/a	n/a	No	No	n/a	n/a

In this study, the *S. haematobium* infection rate decreased significantly in the presence of the WRA. It is possible that infection decreased for a reason not related to the WRA, although to our knowledge, no other relevant community-wide changes took place during the course of the study. It is highly unlikely that individuals received treatment for schistosome infection outside of the praziquantel distribution through our study; praziquantel was difficult to locate in the Atiwa District health clinics near Adasawase at the time of our study, and was occasionally scarce in Accra, the capital of Ghana. The WRA should be tested further in other communities as behavior change may be location-specific. The study shows a biologically-relevant and statistically-significant decrease in *S. haematobium* annual cumulative incidence in a community after installation of a WRA; this decrease was not achieved via MDA alone in the year prior to installing the WRA.
